# Interactive effects of elevated temperature and CO_2_ on two phylogeographically distinct clones of common reed (*Phragmites australis*)

**DOI:** 10.1093/aobpla/pls051

**Published:** 2012-12-20

**Authors:** Franziska Eller, Carla Lambertini, Loc Xuan Nguyen, Luciana Achenbach, Hans Brix

**Affiliations:** Department of Bioscience, Plant Biology, Aarhus University, Ole Worms Alle 1, DK-8000 Aarhus C, Denmark

**Keywords:** Algeria, climate change, Denmark, Mediterranean *Phragmites*, RERAF phytotron, temperate *Phragmites*

## Abstract

The aboveground growth, physiological and biochemical parameters of two clones of the cosmopolitan wetland grass *Phragmites australis,* grown at four treatment combinations of temperature and CO_2_, were investigated to elucidate whether their climate response differed due to inherent differences in their ecological adaptation. The two phylogeographically distinct *P. australis* clones (DK clone, European genetic background; ALG clone, Mediterranean genetic background) were grown for 151 days in phytotrons at 19/12 °C (day/night temperature) and 390 ppm CO_2_, and at elevated temperature (+5 °C) and CO_2_ (700 ppm) with treatment factors alone or in combination. The ALG clone had 2–4 times higher aboveground biomass, higher light-saturated rates of photosynthesis (*P*_max_), maximum electron transport rates (ETR_max_) and Rubisco activity, and higher photosynthetic nitrogen-use efficiency than the DK clone. The DK clone, however, produced more shoots, leaves and side-shoots, and had 9–51 % higher specific leaf area and 15–39 % higher leaf nitrogen concentration than the ALG clone. Although elevated atmospheric CO_2_ alone barely affected the aboveground growth of the two *P. australis* clones, simultaneously elevated temperature and CO_2_ stimulated growth and aboveground biomass. Overall, elevated CO_2_ stimulated photosynthesis, but the clones responded differently to a concomitant increase in CO_2_ and temperature, depending on the phylogeographic background of the plant. The DK clone showed overall stronger responses, and can be considered the more plastic of the two clones with respect to CO_2_ and temperature. Thus, the DK clone may be better adapted to climate change than the ALG clone, at least in the short term.

## Abstract (precis)

One European and one Mediterranean *Phragmites australis* genotype (DK clone and ALG clone, respectively) showed distinct aboveground growth and physiology in response to different treatment combinations of elevated CO_2_ and temperature according to their genetic background. The DK clone was the most responsive clone.

## Introduction

Temperature and atmospheric carbon dioxide (CO_2_) are key environmental parameters affecting plant growth, development and function, and both have changed in the recent past. The concentration of atmospheric CO_2_ has increased from pre-industrial levels of ∼280 ppm to >390 ppm and is predicted to increase further, possibly reaching 700 ppm by the end of the 21st century (Barnola *et al.* 1995; IPCC 2007). Simultaneously, the global average surface temperature is predicted to increase by 0.6–4 °C compared with the beginning of this century (IPCC 2007).

As CO_2_ is a direct resource in the photosynthesis process, elevated CO_2_ is predicted to stimulate plant growth under adequate nutrient supply (Poorter 1993; Wolfe *et al.* 1998). However, the experimental evidence of this is inconclusive (Ainsworth and Long 2005; Rasse *et al.* 2005; Kirschbaum 2011). Long-term exposure of plants to elevated CO_2_ has in some studies resulted in a stimulation of productivity and growth (Wand *et al.* 1999; Lee *et al.* 2010), but in other studies no significant effect has been observed (Milla *et al.* 2006; Kim and Kang 2008). During long-term exposure to elevated CO_2_, leaf photosynthesis may be acclimated to the elevated CO_2_ and hence not elevated, but even down-regulated. However, dependent on the carbon demand and growth capacity, the light-saturated rates of photosynthesis and carbon uptake have also been shown to be stimulated (Ainsworth *et al.* 2003; Tuba and Lichtenthaler 2007). Temperature effects on plant growth can, however, be far more distinct than the effects of CO_2_ (Ojala *et al.* 2002). Since the effects of CO_2_ and temperature on photosynthesis and respiration may counteract each other, the combined effects may differ from those of either factor alone, especially for C_3_ plants (Morison and Lawlor 1999). As climate factors will change concomitantly, understanding their combined effects on a species is crucial for predicting future plant responses.

Wetland plants play a crucial role in ecosystem functioning. Therefore, understanding their responses to changed climate conditions is fundamental for predicting the consequences of global change in aquatic habitats. The common reed, *Phragmites australis* (Cav.) Trin. Ex Steud., is often the dominant plant species of wetland communities. *Phragmites australis* is a perennial, emergent wetland grass that has probably the widest geographical distribution of any flowering plant and is able to acclimate to a wide range of growth conditions (Haslam 1972; Clevering and Lissner 1999). It is found in oligotrophic as well as eutrophic habitats, waterlogged or flooded soils in freshwater and saline wetlands, playing an important role in lake shore protection, habitat provision and wastewater treatment (Clevering 1998; Brix 1999). Differences in ontogeny, shoot morphology, physiological and biochemical processes, and growth characteristics have been reported for several *P. australis* clones native to distinct geographical habitats (Clevering *et al*. 2001; Lessmann *et al.* 2001; Bastlova *et al.* 2004, 2006; Hansen *et al.* 2007; Lambertini *et al.* 2012). The species generally shows high phenotypic plasticity when introduced to an environment distinct from its natural habitat (Lessmann *et al.* 2001) and even appears as a vigorous invader, as seen in North America, where an aggressive lineage has invaded salt marshes along coastal New England, threatening marsh habitat quality for wildlife (Saltonstall 2002). Acclimation and plasticity in response to changing environments and climate indicate a high potential of *P. australis* to sustain its fitness under future climate change scenarios. However, it is unknown how growth and photosynthesis of distinct *P. australis* clones are responding to future climate change scenarios, i.e. the simultaneously elevated atmospheric CO_2_ concentration and temperature.

The aim of this study was to assess the effects of elevated temperature and CO_2_ alone and in combination on aboveground growth, physiological and biochemical parameters of two contrasting *P. australis* clones. The clones originated from two different phylogeographic lineages, namely the European temperate *P. australis* gene pool and the Mediterranean gene pool (Lambertini *et al.* 2006, 2012). The two lineages were shown to be genetically distinct in the aforementioned studies, and their confined latitudinal distribution ranges suggest different ecological adaptations. Therefore, the two clones may respond distinctly to climate change and the single climate factors may affect each clone in a dissimilar manner. We hypothesized that the two clones would respond differently to temperature and CO_2_ due to their inherent differences in ecological adaptation. Moreover, since *P. australis* has a strong carbon sink capacity, i.e. a large carbon uptake capability due to its large vegetative biomass production and development of tillers and side-shoots, we hypothesized that elevated CO_2_ would stimulate plant growth under non-limiting nutrient conditions.

## Methods

### Experimental treatments

The response of two clones of common reed (*P. australis*) to elevated temperature (+5 °C) and elevated CO_2_ (700 ppm) was studied in a phytotron system with treatment factors operating separately or in combination. The treatments were set up in four physically and electronically separated gas-tight walk-in chambers (6 m width, 4 m length, 3.1 m height) of the climate phytotron Risø Environmental Risk Assessment Facility (RERAF, DTU, Denmark).

Each of the chambers was equipped with separate ventilation systems and individual control of light, temperature, humidity and CO_2_. Two fans mounted on opposite sides in each chamber ensured air mixing. A 16/8-h day/night light regime was provided by high-pressure mercury and halogen lamps supporting the natural sunlight entering through the transparent glass roof and outside wall, resulting in a daytime irradiance of ∼400–600 µmol m^−2^ s^−1^ (photosynthetic photon flux density, PPFD) at the base of the plant pots. Sunrise and sunset were simulated during the first and last hour of the day-light regime by a gradual change in the light intensity. The relative air humidity (RH) was controlled at 65/80 % (day/night).

The four treatment combinations were (i) ‘Ambient’, (ii) ‘Temp’ (elevated temperature, +5 °C), (iii) ‘CO_2_’ (elevated [CO_2_], ∼700 ppm) and (iv) ‘Temp + CO_2_’ (elevated temperature and CO_2_). The ‘Ambient’ treatment, which served as a control, simulated present Northern European early summer season conditions with 19/12 °C day/night temperatures and ∼390 ppm CO_2_. The measured concentrations of CO_2_ in the growth chambers were slightly above the intended set-point levels in the ‘Ambient’ and ‘Temp’ treatments, especially during the night. This may be a consequence of negligibly increased respiration from the plants and soil.

### Plant material and growth conditions

The two clones used in this study were chosen from a large collection of live *P. australis* clones, kept in a common environment at Aarhus University, Denmark (56°13′N; 10°07′E), for at least 6 years prior to this study. A clone from a coastal stand close to Aarhus, Denmark (56°12′N; 10°29′E) (DK clone) and a clone from an oasis in the Sahara desert close to Guebbour, Algeria (28°29′N; 6°41′E) (ALG clone) were chosen for this study. The two clones were selected based on their phylogeographic relationship. The DK clone possesses the alleles of European temperate *P. australis*, whereas the ALG clone belongs to the Mediterranean gene pool of *P. australis*. The two phylogeographic groups within *P. australis* were identified by amplified fragment length polymorphisms (Lambertini *et al.* 2006) and chloroplast DNA sequences (Lambertini *et al.* 2012) and described as temperate European and Mediterranean *Phragmites* (Lambertini *et al.* 2012). The Mediterranean *Phragmites* extends in its distribution throughout Southern Europe, North Africa and the Middle East. Differences between the two clones are therefore driven by different evolutionary pressures in their native distribution ranges.

Clones were propagated by layering of shoots horizontally in water for 35 days to initiate adventitious shoot growth at the stem nodes. When adventitious shoots were 150–200 mm tall and had developed roots, the stems were cut at both sides of the nodes and the resulting replicate plants planted in 3.5-L pots containing a commercial peat. The plants were then acclimated for 3 weeks in one of the growth chambers at the ‘Ambient’ treatment conditions, as described above. Plants were fertilized weekly with 0.5 L of a nutrient solution containing a commercial fertilizer with N–P–K values of 6–1–5 (Substral^®^; The Scotts Company, Nordics A/S, Glostrup, Denmark). Additional micronutrients were added to the nutrient solution from a micronutrient stock solution (Pioner Mikro Plus with Fe; Brøste, Lyngby, Denmark) in concentrations of (in µM) 0.02 B; 2.2 Cu; 24 Fe; 9.1 Mn; 0.5 Mo; 2.8 Zn. Additional iron (Fe^2+^) was added to the nutrient solution as Fe(II)SO_4_ (∼0.59 mM) right before every fertilization. Between fertilizations, the plants were watered with tap water. Tables holding the plants were rotated within the growth chambers weekly and between the chambers bi-weekly, to avoid undesired chamber effects.

The experimental set-up was a 2 × 2 × 2 factorial design with the factors being clone (DK vs. ALG), temperature (19/12 vs. 24/17 °C) and CO_2_ concentration (390 vs. 700 ppm). Ten replicates of each clone were used in each of the four treatments (*n* = 10 for all measured parameters). One replicate of the DK clone in the ‘Temp’ treatment died and hence was removed from all statistical analyses (*n* = 9 for all parameters from the ‘Temp’ treatment, DK clone). The experiment was run for 151 days after the initial acclimatization period.

### Plant aboveground biomass and growth parameters

Numbers of shoots and leaves per plant were counted at the start of the experiment (Day 0) and after 52 days when the aboveground biomass was harvested. The number of leaves per plant included all fully developed leaves. Absolute shoot and leaf production rates were determined as the difference between the final and the initial number of shoots and leaves, respectively, divided by the growth time (52 days). The cumulative length of all shoots per plant was measured at harvest, and the number of side-shoots developed at the shoot nodes was counted. All plant fractions were dried to a constant dry mass at 80 °C in a ventilated oven to determine the final aboveground biomass. The pots with the cut stems containing the roots and rhizomes were kept in the treatment chambers for an additional period of 99 days, and were watered and fertilized as described above. The new shoots that developed from the rhizome system were used for photosynthetic measurements after 95–99 days of re-growth.

### Photosynthetic measurements

Photosynthesis of the third or fourth youngest leaf of one shoot per replicate was measured with an ADC LCA-4 infrared gas analyser (IRGA) equipped with a Leaf Microclimate Control System (ADC BioScientific Ltd, Hoddesdon, UK). The leaf chamber was air-conditioned at the respective growth temperature and placed on a tripod to ensure stability during readings. The leaf chamber was supplied with air from inside the growth chamber drawn from a height of 3 m above the floor at a flow rate of 300 µmol s^−1^ by the built-in air pump of the IRGA. Light was supplied from a white halogen source (Portable Light Unit, PLU-002, ADC BioScientific Ltd, Hoddesdon, UK) at an irradiance (PPFD) of ∼1800 µmol m^−2^ s^−1^. The maximum light-saturated rate of photosynthesis (*P*_max_) was estimated as the average of five readings that were logged after a stable CO_2_ assimilation rate was reached.

### Chlorophyll fluorescence

Chlorophyll fluorescence was measured with a portable fluorometer (PAM-2000; Walz Mess- und Regeltechnik GmbH, Effeltrich, Germany). Measurements were performed at the third or fourth youngest leaf of one shoot per replicate using leaf-clip holders. The potential quantum yield of photosystem II (PS II) (*F*_v_/*F*_m_) in 20-min dark-incubated leaves (darkened by a leaf clamp) was measured using the Saturation Pulse method of the PamWin Windows Software for the PAM-2100 Chlorophyll Fluorometer (Software V 1.17; Walz GmbH, Effeltrich, Germany). Rapid light curves were measured using a pre-installed software routine where actinic irradiance was increased in 10 steps from 11 to 218 µmol photons m^−2^ s^−1^ for a duration of 20 s per step. The calculated electron transport rate (ETR) was plotted against the actinic irradiance (*I*). To determine the maximum rate of electron transport (ETR_max_) and the effective quantum yield (*α*), the curves were fitted to the photosynthesis–irradiance model of Harrison and Platt (1986) corrected for the absence of photoinhibition according to Ralph and Gademann (2005), according to the following equation:
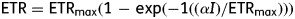



### Photosynthetic pigments and specific leaf area

The third or fourth youngest leaf, which was also used for measurement of photosynthesis, was photocopied and the area determined by ImageJ v. 1.43, a Java-based image analysis software (Abramoff *et al.* 2004). The dry mass after lyophilization of the leaf that had been photocopied was determined prior to pigment analysis, and used to calculate the specific leaf area (SLA; the ratio of leaf area to leaf dry mass). Concentrations of total chlorophylls (Chl_a_ and Chl_b_) and total carotenoids (xanthophylls and carotenes; C_(x+c)_) of the same leaf used for photosynthesis and SLA measurement were analysed by photospectrometry after extraction in 96 % ethanol according to Lichtenthaler (1987).

### Tissue nitrogen and carbon, and photosynthetic nitrogen-use efficiency

Total carbon (C) and nitrogen (N) were analysed for 1.5–3.5 mg of ground leaf material with a CHN analyser (Fisons Instruments, Model NA2000, Rodano, MI, Italy) and expressed on a % dry mass basis. Photosynthetic nitrogen-use efficiency (PNUE) was calculated as the ratio of *P*_max_ to leaf N concentration.

### Rubisco activity

Total activity of ribulose-1.5-bisphosphate carboxylase/oxygenase (Rubisco) was analysed in the third or fourth youngest leaves of two or three shoots of the same age in each replicate (all leaves from the chosen shoots pooled from each replicate plant). Entire leaves were cut in the light and frozen immediately in liquid nitrogen. The frozen plant material was ground in a mortar containing liquid nitrogen. Approximately 0.1 g of homogenate was further ground in a chilled mortar with 5 mL of extraction buffer containing 50 mM bicine (pH 8), 0.1 mM EDTA-Na_2_, 10 mM MgCl_2_, 5 mM dithiothreitol (DTT), 10 mM isoascorbate and 2 % (w/v) polyvinylpyrrolidone. Rubisco activity was determined in an assay solution consisting of 50 mM bicine (pH 8), 0.1 mM EDTA-Na_2_, 10 mM MgCl_2_, 5 mM DTT and 19.25 mM NaH^14^CO_3_. After the addition of ground extract, vials were incubated for 5 min before activation with 0.5 mM RuDP (ribulose 1,5-diphosphate). The reaction was stopped after 60 s with 6 M HCl. All reactions were carried out at 17 °C in a total volume of 500 µL using 6-mL vials. Samples were dried at 60 °C for 24 h and afterwards re-dissolved in two drops of 6 M KOH and 1.2 mL of ultrafiltered water. The amount of radioactive decay energy was measured by liquid scintillation counting (Liquid Scintillation Analyzer, Tri-CARB 2100 TR; Packard, Meriden, CT, USA). The concentration of Chl_(a+b)_ in the extract was analysed using 96 % ethanol (µg Chl_(a+b)_ mL^−1^ ground extract) and used together with SLA to convert the Rubisco activity to a leaf area basis.

### Statistical analyses

Data were analysed by the General Linear Model procedure using Type III sum of squares with the software Statgraphics Centurion XVI (Statpoint Technologies, Inc., Warrenton, VA, USA). Data were tested for variance homogeneity using Levene's test and, if necessary, log-transformed to ensure homogeneity of variances. The third-order interaction term was suppressed.

A rotated factor analysis was conducted on all results. The number of factors was restricted to three, and the factor scores after Varimax rotation were used for interpretation of the data variability.

## Results

### Plant growth and aboveground biomass

**Fig. 1:**
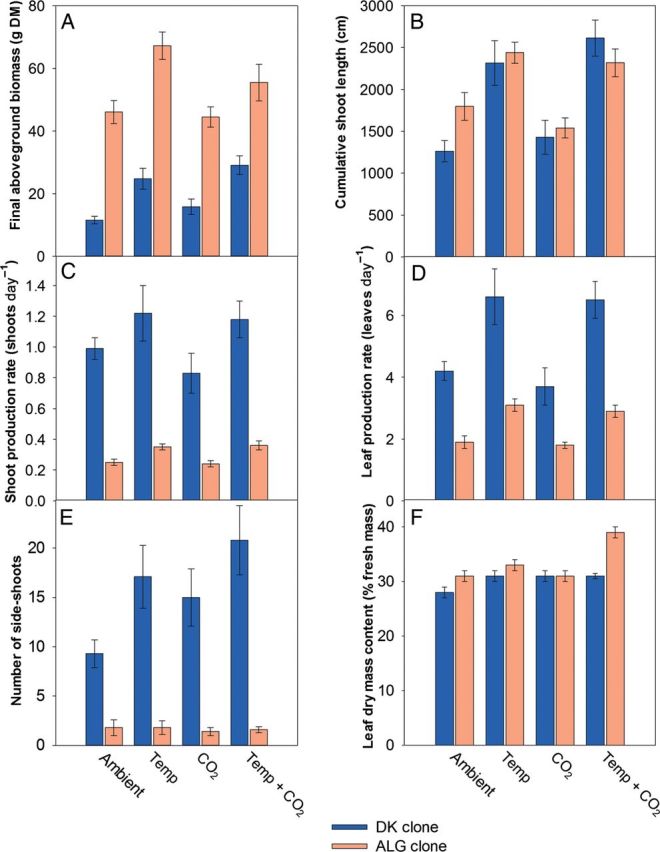
Mean (±S.E.) final aboveground biomass (A), cumulative shoot length (B), shoot production rate (C), leaf production rate (D), final number of side-shoots (E) and leaf dry mass content (F) of two distinct *P. australis* clones (DK clone and ALG clone) grown at four different treatments (‘Ambient’; ‘Temp’, elevated temperature, +5 °C; ‘CO_2_’, elevated CO_2_, ∼700 ppm; ‘Temp + CO_2_’, elevated CO_2_ and temperature +5 °C).

The two clones differed significantly in both growth pattern and morphology (Table 1). The ALG clone had developed 2–4 times higher final aboveground biomass than the DK clone (Fig. 1A). Temperature mainly affected the final aboveground biomass of the DK clone, which was approximately twice as high at elevated temperature compared with ambient temperature (clone × temperature interaction). Both clones also had up to 50 % (ALG clone) and 83 % (DK clone) longer shoots and up to 100 % (ALG clone) and 50 % (DK clone) more shoots as well as up to 63 % (ALG clone) and 76 % (DK clone) more leaves at elevated temperature (Fig. 1B–D). The ALG clone consistently had 3–4 times lower shoot density, 5–13 times fewer side-shoots, and produced ∼55 % fewer leaves than the DK clone (Fig. 1C–E). The cumulative shoot length, however, did not differ significantly between the clones (Fig. 1B).

**Table 1:** **Summary of three-way ANOVA (F-ratios; d.f. total=78) showing the effects of clone (ALG vs. DK), temperature (19/12 vs. 24/17 °C) and CO_2_ (390 vs. 700 ppm), and their interactions on growth and physiological parameters of two *P. australis* clones.** *P*_max_, maximum light-saturated rate of photosynthesis; *F*_v_/*F*_m_, potential quantum yield of PS II; *α*, effective quantum yield; ETR_max_, maximum rate of electron transport; SLA, specific leaf area; C_(x+c)_, total carotenoids; C_(a+b)_/C_(x+c)_, ratio of chlorophyll a and b to total carotenoids; PNUE, photosynthetic nitrogen-use efficiency; d.f., degrees of freedom. Significance level (in bold): *P < 0.05; **P < 0.01; ***P < 0.001.

Parameter	Source of variation					
	Clone (d.f. = 1)	Temperature (d.f. = 1)	CO_2_ (d.f. = 1)	Clone × temperature	Clone × CO_2_	Temperature × CO_2_
Final aboveground biomass	100.1***	23.1***	0.0	5.1*	1.5	0.1
Final cumulative shoot length	0.9	53.6***	0.0	2.7	2.95	0.3
Number of side-shoots	146.5***	2.3	0.1	2.9	1.6	0.2
Shoot production rate	146.2***	13.9***	1.0	0.2	0.9	1.2
Leaf production rate	37.3***	23.4***	1.1	0.3	0.3	0.7
Leaf dry mass content	27.9***	24.8***	12.8***	8.2**	0.1	1.3
*P*_max_	43.1***	8.8**	113.3***	0.4	0.9	1.8
*F*_v_/*F*_m_	41.1***	0.4	0.3	0.4	13.2***	15.4***
α	1.8	6.8*	54.6***	0.3	6.1*	7.6**
ETR_max_	8.2**	1.5	2.5	0.8	0.1	3.4
Rubisco activity	9.5**	22.7***	7.8**	1.0	3.1	0.2
SLA	94.9***	0.9	16.3***	0.5	7.9**	0.1
Chl_a_	3.5	10.1**	1.98	0.0	1.8	4.8*
Chl_b_	3.9	5.2*	5.7*	2.5	2.0	1.1
Chl a/b ratio	0.3	9.8**	26.0***	54.0***	0.0	15.1***
C_(x+c)_	3.5	16.4***	0.7	1.2	0.6	10.0**
C_(a+b)_/C_(x+c)_	0.3	2.8	11.4**	17.6***	3.2	4.6*
PNUE	23.9***	0.1	46.7***	2.6	9.0**	7.9**
Leaf N	50.7***	32.5***	2.7	0.6	3.3	3.7
Leaf C	62.9***	0.1	0.4	0.3	0.5	9.6**

The CO_2_ concentration did not affect any growth or biomass parameter, except for the leaf dry mass content, which was higher at elevated CO_2_. The leaf dry mass content was also affected by a clone × temperature interaction, as the ALG clone had 5–26 % higher leaf dry mass content at elevated temperature, compared with ambient temperature (Fig. 1F).

### Photosynthesis, chlorophyll fluorescence and Rubisco activity

The two clones differed significantly in their photosynthetic parameters (Table 1). The ALG clone had significantly higher *P*_max_, ETR_max_ and Rubisco activities than the DK clone (14–153 %, 13–46 % and 26–87 % within the treatments, respectively) (Table 2). The *P*_max_ and Rubisco activities for both clones were similarly affected by temperature and CO_2_. Thus, elevated temperature tended to decrease *P*_max_ by 56 % (DK clone at ambient CO_2_) and by up to 14 % (ALG clone), and the Rubisco activity by up to 37 % and 48 % (ALG clone, and DK clone, respectively). Elevated CO_2_ tended to increase *P*_max_ by up to 77 % (ALG clone) and 226 % (DK clone), and the Rubisco activities by 13 % (ALG clone at ambient temperature) and up to 107 % (DK clone). With respect to *α* and *F*_v_/*F*_m_, the DK clone responded more to CO_2_ than the ALG clone and had up to 4 % and 14 % higher values (for *F*_v_/*F*_m_ and *α*, respectively) at elevated than at ambient CO_2_, as compared with the ALG clone, which merely differed by 1 % and up to 7 % (for *F*_v_/*F*_m_ and *α*, respectively; clone × CO_2_ interaction). For both parameters, a CO_2_ × temperature interaction was also found, where a temperature effect only occurred at ambient CO_2_. Here, *α* was highest at ambient temperature, whereas *F*_v_/*F*_m_ was highest at elevated temperature (Table 2).

**Table 2:** **Photosynthetic parameters (mean values ± S.E.) of two distinct *P. australis* clones grown at different CO_2_ concentration and temperature treatments.** The four treatments were ‘Ambient’; ‘Temp’, elevated temperature; ‘CO_2_’, elevated CO_2_; ‘Temp + CO_2_’, elevated temperature and CO_2_.

Parameter	Clone	Treatment			
		‘Ambient’	‘Temp’	‘CO_2_’	‘Temp + CO_2_’
*P*_max_ (µmol m^−2^ s^−1^)	DK	14.1 ± 1.0	6.2 ± 0.8	19.3 ± 0.9	20.2 ± 1.7
	ALG	16.1 ± 1.5	15.7 ± 0.9	28.5 ± 1.5	24.4 ± 1.3
*F*_v_/*F*_m_	DK	0.80 ± 0.00	0.82 ± 0.00	0.83 ± 0.00	0.82 ± 0.00
	ALG	0.80 ± 0.00	0.81 ± 0.00	0.80 ± 0.00	0.79 ± 0.01
α	DK	0.311 ± 0.004	0.291 ± 0.003	0.326 ± 0.002	0.328 ± 0.003
	ALG	0.308 ± 0.002	0.299 ± 0.004	0.318 ± 0.005	0.316 ± 0.005
ETR_max_ (µmol e^−^ m^−2^ s^−1^)	DK	89 ± 6	66 ± 6	80 ± 4	97 ± 11
	ALG	112 ± 13	85 ± 6	117 ± 22	110 ± 10
Rubisco activity (µmol m^−2^ s^−1^)	DK	8.9 ± 0.7	4.6 ± 0.5	10.8 ± 1.2	9.5 ± 0.9
	ALG	12.1 ± 1.7	8.7 ± 1.0	13.7 ± 1.2	8.6 ± 0.4

### SLA and pigment concentrations

**Fig. 2:**
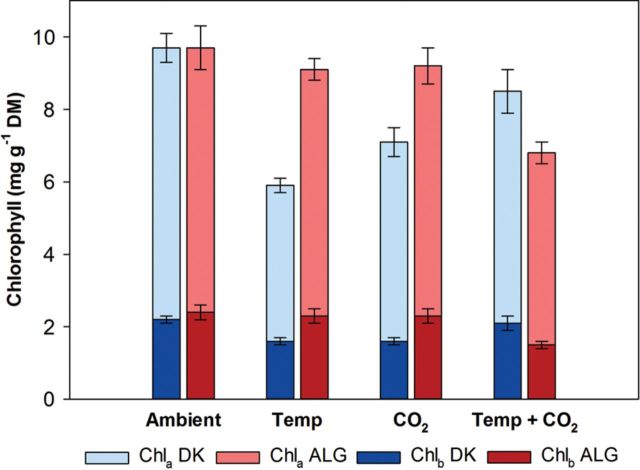
Mean (±S.E.) chlorophyll concentrations (Chl_a_ and Chl_b_) in leaves of two distinct *P. australis* clones (DK clone and ALG clone) grown at the four different treatments.

There was no significant difference between the two clones for the photosynthetic pigments or their ratios (Table 1). A CO_2_ × temperature interaction affected Chl_a_, the Chl a/b ratio, C_(x+c)_ and the ratio of total chlorophyll to carotenoids (C_(a+b)_/C_(x+c)_), as a temperature response was only seen at ambient CO_2_. Here, Chl_a_, the Chl a/b ratio and C_(x+c)_ were highest at the ambient temperature whereas C_(a+b)_/C_(x+c)_ was highest at the elevated temperature (Fig. 2; Table 3). Also Chl_b_ was highest at ambient temperature (Fig. 2). For the Chl a/b ratio and C_(a+b)_/C_(x+c)_, clones responded distinctly within the CO_2_ treatments, and the DK clone showed the strongest responses to temperature (clone × temperature interaction). At ambient CO_2_, the Chl a/b ratio of the DK clone was 26 % higher at ambient than at elevated temperature, whereas the ALG clone showed no temperature response. At elevated CO_2_, both clones differed in their temperature responses by 13 %, where the highest Chl a/b ratio was at ambient temperature for the DK clone and at elevated temperature for the ALG clone. C_(a+b)_/C_(x+c)_ of the DK clone was higher at elevated temperature (11 % and 15 % at ambient and elevated CO_2_, respectively), whereas C_(a+b)_/C_(x+c)_ at elevated temperature for the ALG clone increased by 4 % at ambient CO_2_ and decreased by 15 % at elevated CO_2_ (Table 3). The DK clone had 9–51 % higher SLA than the ALG clone (Table 3). The ALG clone responded most to CO_2_ and had up to 32 % higher SLA at ambient CO_2_ compared to elevated CO_2_ (clone × CO_2_ interaction).

**Table 3:** The SLA, PNUE, photosynthetic pigments, leaf N and C concentration (mean values ± S.E.) of two distinct *P. australis* clones grown at the different CO_2_ concentration and temperature treatments.

Parameter	Clone	Treatment			
		‘Ambient’	‘Temp’	‘CO_2_’	‘Temp + CO_2_’
SLA (m^2^ kg^−1^ DM)	DK	20.8 ± 0.4	20.1 ± 0.6	19.0 ± 0.6	21.0 ± 0.5
	ALG	16.7 ± 0.3	18.4 ± 0.8	15.4 ± 0.6	13.9 ± 0.5
PNUE (µmol C g^−1^ N s^−1^)	DK	7.2 ± 0.5	3.5 ± 0.5	9.2 ± 0.7	15.7 ± 2.1
	ALG	8.9 ± 1.0	11.3 ± 1.0	14.0 ± 1.0	13.9 ± 1.4
Chl a/b ratio	DK	3.37 ± 0.04	2.71 ± 0.09	3.45 ± 0.07	3.11 ± 0.06
	ALG	3.06 ± 0.11	3.05 ± 0.06	3.10 ± 0.06	3.52 ± 0.04
C_(x+c)_ (mg g^−1^ DM)	DK	1.57 ± 0.06	0.87 ± 0.04	1.19 ± 0.06	1.25 ± 0.10
	ALG	1.49 ± 0.16	1.29 ± 0.05	1.37 ± 0.08	1.21 ± 0.05
C_(a+b)_/C_(x+c)_	DK	6.2 ± 0.1	6.9 ± 0.2	5.9 ± 0.2	6.8 ± 0.1
	ALG	6.7 ± 0.3	7.0 ± 0.2	6.7 ± 0.2	5.7 ± 0.2
Leaf N (% DM)	DK	4.05 ± 0.05	3.67 ± 0.10	4.01 ± 0.12	2.95 ± 0.22
	ALG	3.09 ± 0.17	2.64 ± 0.13	3.21 ± 0.17	2.56 ± 0.21
Leaf C (% DM)	DK	43.5 ± 0.1	44.9 ± 0.3	44.5 ± 0.3	43.2 ± 0.8
	ALG	46.0 ± 0.1	46.1 ± 0.2	46.3 ± 0.4	45.8 ± 0.2

### PNUE, N and C concentration

The DK clone had 15–39 % higher leaf N concentration than the ALG clone, but the ALG clone contained more carbon (3–6 %) than the DK clone (Table 3). Plants had 19–23 and 11–33 % (ALG clone and DK clone, respectively) higher N concentration at ambient temperature compared with elevated temperature. The carbon concentration of both clones was affected by a CO_2_ × temperature interaction. When grown at elevated temperature, both clones had higher C concentration at ambient CO_2_. At ambient temperature, elevated CO_2_ resulted in higher C concentrations.

The ALG clone had up to three times higher PNUE than the DK clone, but the DK clone showed the strongest CO_2_ response by having 28–349 % higher PNUE at elevated CO_2_, as opposed to the ALG clone (23–57 %; clone × CO_2_ interaction). Also the PNUE was affected by a CO_2_ × temperature interaction, as the response of PNUE to CO_2_ mostly occurred at elevated temperature, with the PNUE being highest at elevated CO_2_.

### Factor analysis

All measured parameters were simplified into three major factors describing 78.8 % of the variation in the responses of the plants to the treatments (Table 4).

**Table 4:** Factor loadings for Varimax-rotated factor analysis of measured parameters of two distinct *P. australis* clones grown at different CO_2_ concentration and temperature treatments.^a^

	Factor 1	Factor 2	Factor 3
Eigenvalue	4.80	3.50	2.91
Proportion of variance (%)	33.8	24.6	20.4
Cumulative proportion of variance (%)	33.8	58.4	78.8
*Parameter*			
SLA	**0.833**	0.359	0.061
Shoot production rate	**0.797**	−0.148	0.269
Number of side-shoots	**0.784**	−0.151	0.082
Leaf production rate	**0.647**	−0.153	0.553
*P*_max_	**−0.619**	0.295	0.041
Leaf dry mass content	**−0.555**	−0.552	0.246
PNUE	**−0.504**	0.378	0.278
Leaf C	**−0.427**	−0.051	0.082
*F*_v_/*F*_m_	**0.422**	−0.161	−0.020
ETR_max_	**−0.367**	0.200	0.044
Chl a/b ratio	**−0.327**	−0.016	−0.325
Chl_a_	−0.086	**0.985**	0.059
Chl_b_	0.007	**0.929**	0.181
C_(x+c)_	−0.146	**0.884**	−0.126
Rubisco activity	−0.302	**0.527**	−0.058
α	−0.106	**0.162**	−0.130
Final cumulative shoot length	0.127	−0.170	**0.889**
Final aboveground biomass	−0.410	0.050	**0.835**
Leaf N	0.442	0.040	**−0.708**
C_(a+b)_/C_(x+c)_	0.194	0.279	**0.455**

^a^Variables with high loadings are shown in bold.

**Fig. 3:**
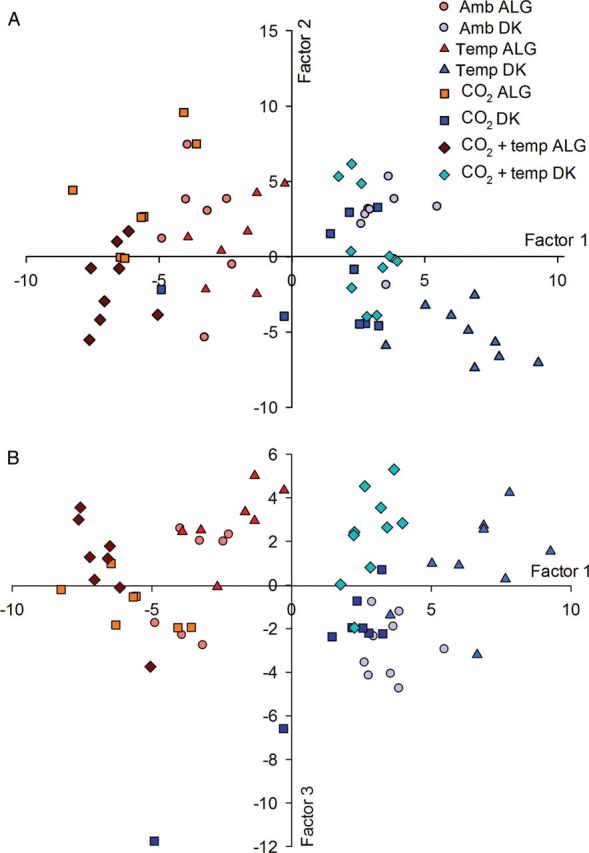
**Factor scores of Factor 1 against Factor 2 (A) and Factor 1 against Factor 3 (B) of a rotated factor analysis of two distinct *P. australis* clones (DK clone and ALG clone) grown at the four different treatments.** Factor 1 has high loadings for shoot growth, photosynthesis and leaf C content; Factor 2 has high loadings for pigments, Rubisco activity and yield *α*; Factor 3 has high loadings for the final aboveground biomass and cumulative shoot length, leaf N concentration and C_(a+b)_/C_(x+c)_.

The first factor explained 33.8 % of the variation and had high positive loadings for SLA, the shoot and leaf production rates, the number of side-shoots and *F*_v_/*F*_m_, and high negative loadings for the leaf dry mass content, leaf C content, PNUE, *P*_max_, ETR_max_ and the Chl a/b ratio. Factor 2 explained an additional 24.6 % of the variation and had high positive loadings for Chl_a_ and Chl_b_, C_(x+c)_, Rubisco activity and *α*. Factor 3 explained an additional 20.4 % of the variation. It had high negative loadings for the leaf N concentration and positive loadings for the final aboveground biomass and cumulative shoot length as well as C_(a+b)_/C_(x+c)_. Figure 3 shows the distribution of the clones according to the experimental treatments within the first two factors (Fig. 3A) and within Factor 1 against Factor 3 (Fig. 3B). Both clones were clearly separated from each other according to Factor 1, reflecting their differences in growth-related parameters as well as parameters associated with photosynthetic performance, most of which also showed significant differences for ‘clone’ in the analysis of variance (ANOVA) (Table 1). The ALG clone was hardly affected by treatment along Factor 2, which had high loadings for pigments, quantum yield *α* and Rubisco. The DK clone, however, was affected by the ‘Temp’ treatment, which was separated on Factors 1 and 2 from the other treatments. Mainly the ALG clone, and to a lesser extent also the DK clone, were separated along Factor 1 according to CO_2_ in the treatments, involving either ambient or elevated CO_2_ (Fig. 3A). Factor 3, which had the highest loadings for the final aboveground biomass and shoot length, separated the ‘Temp’ and the ‘CO_2_ + temp’ treatments from the ‘CO_2_’ treatment. For the ALG clone, the ‘Ambient’ treatment was located in between these treatments, whereas for the DK clone, the ‘Ambient’ treatment was separated largely from the ‘Temp’ and ‘CO_2_ + temp’ treatment. Hence, Factor 3 mainly separated the DK clone according to treatments involving either elevated or ambient temperature (Fig. 3B).

## Discussion

The aim of this study was to evaluate to what extent two phylogeographically distinct clones of *P. australis* respond to elevated temperature and CO_2_ concentration, as single abiotic factors or in combination. We hypothesized that the two clones would respond differently to a change in these climatic parameters, due to their genetically inherent differences in adaptation to distinct climatic origins.

It has been postulated that the genetic variation in *P. australis* has arisen in response to the latitude of its geographical origin (Clevering *et al.* 2001; Lessmann *et al.* 2001; Bastlova *et al.* 2004). Clevering *et al.* (2001) showed that *P. australis* originating from higher latitudes, such as the DK clone, developed a higher number of shoots and leaves, and larger SLA compared with genotypes from lower latitudes, such as the ALG clone. In the present study, the DK clone and the ALG clone not only differed in most plant traits measured, but also responded differently to CO_2_ and temperature for many of the parameters, as was hypothesized. The ALG clone had the highest aboveground biomass development but the aboveground biomass of the DK clone increased more than that of the ALG clone in response to elevated temperature. Temperature affected the Chl a/b ratio as well as C_(a+b)_/C_(x+c)_ more in the DK clone than in the ALG clone, which is visualized in the factor analysis (Fig. 3A). The DK clone also demonstrated a stronger response to elevated CO_2_ with increased PNUE, *α* and *F*_v_/*F*_m_, whereas the ALG clone only responded to elevated CO_2_ with a lower SLA, as shown in the ANOVA interactions. Hence, the DK clone showed an overall stronger response to future climate change factors than the ALG clone and can therefore be considered the more plastic of the two clones with respect to atmospheric CO_2_ and temperature. Higher phenotypic plasticity of the DK clone with respect to temperature has been shown previously, when compared with the ALG clone (Eller and Brix 2012).

An often observed response to CO_2_ enrichment is acclimation of the initial stimulation of photosynthesis and down-regulation of the Rubisco activity due to feedback inhibition caused by the accumulation of carbohydrates (Paul and Driscoll 1997). In C_3_ plants, the acclimation of the photosynthetic capacity to elevated CO_2_ after long-term exposure is often seen as a decrease in the maximum carboxylation rate of Rubisco (*V*_cmax_) and the ETR_max_. However, despite this down-regulation of the photosynthetic capacity, the net carbon uptake can be enhanced by elevated CO_2_ (Leakey *et al.* 2009). Contrary to the growth and aboveground biomass parameters in the present study, most of the analysed photosynthetic parameters and pigments, as well as the PNUE and C content, were affected by CO_2_ or interactions of CO_2_ with either clone or temperature. This physiological response to CO_2_ could be expected, since *P. australis* generally uses the C_3_ photosynthetic pathway (Antonielli *et al.* 2002), where carbon uptake is saturated at a much higher internal CO_2_ concentration than in C_4_ plants, for which elevated atmospheric CO_2_ has no direct effect on carbon uptake under favourable conditions (Leakey *et al.* 2009). At ambient temperature, the two *P. australis* clones showed higher Rubisco activities at elevated CO_2_ compared with ambient CO_2_ concentration. Plants with Rubisco-limited photosynthetic capacity, such as the plants in this study, have a larger potential for CO_2_ stimulation of photosynthesis because the increased CO_2_ both increases Rubisco carboxylation rates and depresses photorespiration (Ainsworth and Rogers 2007). Since no down-regulation of Rubisco activity, ETR_max_ or *α* was observed in our study, photosynthesis of the two clones did not acclimate to the increased CO_2_ concentration. A possible reason for this is that the plants in the present study had sufficient nutrients. Acclimation of photosynthesis has been reported to be more marked in nitrogen-limited plants exposed to elevated CO_2_ (Riviere-Rolland *et al.* 1996). This may be due to nitrogen allocation away from Rubisco, mainly towards root growth to increase nutrient acquisition (Wolfe *et al.* 1998).

Contrary to our expectations, we did not observe any increase in the aboveground biomass or the other measured growth parameters of the two clones grown at elevated CO_2_ in the present study, despite the fact that *P*_max_, *α*, Rubisco activity and PNUE were stimulated in the ‘CO_2_’ treatment. Kirschbaum (2011) pointed out that an increase in photosynthesis will not necessarily lead to an equivalent enhancement in growth, but that the potentially gained carbon may be utilized differently. The transformation of photosynthetic carbon gain into aboveground growth might be inefficient due to a lack of shoot sink capacity (i.e. tissues that can incorporate the excess carbon) and the influence of other limiting factors, such as a shortage of water, nutrient or light. In the present study, only aboveground biomass was investigated. The general observation from studies on root growth under elevated CO_2_ is that root growth is stimulated, resulting in an increase in the root to shoot ratio (Norby 1994; BassiriRad *et al.* 2001). Since we did not quantify the belowground biomass, it cannot be ruled out that elevated CO_2_ in the present experiment had a stimulating effect on root growth or that a surplus of assimilated carbon was stored in rhizomes. Under field conditions, overwintering rhizomes serve as carbon storage to support shoot growth in the following spring. This might lead to an underestimation of the response to elevated CO_2_ in short-term experiments (Norby 1994). The lack of an aboveground biomass response to elevated CO_2_ in the ‘CO_2_’ treatment observed in the present study cannot be explained by insufficient fertilization, since plants in the ‘Temp + CO_2_’ treatment reached a higher biomass under the same nutrient availability.

Enhanced growth of shoot biomass (Daepp *et al.* 2000; Rasse *et al.* 2005) as well as total plant biomass (Wand *et al.* 1999; Poorter and Perez-Soba 2001) has often been observed in plants grown at elevated CO_2_, especially C_3_ plants, at least in the short term and at non-limiting nutrient conditions. However, for the *P. australis* clones, investigated here it was only in combination with higher growth temperature that elevated CO_2_ resulted in increased aboveground biomass. In the ‘Temp + CO_2_’ treatment, the increased availability of respiratory substrates and increased enzyme capacity due to the elevated temperature presumably resulted in higher respiration rates, which in combination with the higher rates of photosynthesis could result in higher growth rates (Atkin and Tjoelker 2003).

In the present study, the two *P. australis* clones responded differently to growth temperature with respect to final aboveground biomass, with the DK clone being more responsive to temperature than the ALG clone. Plant species with a large geographic distribution, such as *P. australis*, may have different optimum temperatures and acclimation potentials (Berry and Björkman 1980). The low *P*_max_, *α*, ETR_max_ and Rubisco activity of the two *P. australis* clones in the ‘Temp’ treatment suggest high rates of photorespiration at the elevated temperature, due to the enhanced oxygenation reaction of Rubisco impacting the C_3_ pathway of photosynthesis (Berry and Björkman 1980). However, the fairly constant *F*_v_/*F*_m_ ratio, which is close to 0.8 in healthy vascular plants (Björkman and Demmig 1987), indicates that the plants in the ‘Temp’ treatment were not stressed. Moreover, the leaves were acclimated to adequate light availability as judged from the Chl a/b ratios, which were generally higher than those of typical shade leaves (Demmig-Adams 1998; Lichtenthaler *et al.* 2007). Hence, despite the slightly lower photosynthetic parameters at elevated temperature, the growth and aboveground biomass were not negatively affected. A similar response has previously been observed for the two clones (Eller and Brix 2012).

Carbon dioxide and temperature interact in their effects on plants. Elevated CO_2_ can counteract the negative effects of high temperature (Lee 2011) but high temperatures may also offset the stimulating effect of elevated CO_2_ (Clausen *et al.* 2011). Although interactions between temperature and CO_2_ occurred for some photosynthetic parameters, the pigment ratios, C_(x+c)_, Chl_a_ and the leaf C content, no interaction effects were seen on growth and biomass. All growth and biomass traits were only affected by temperature, except for the leaf dry mass content, which was highest at elevated CO_2_, probably due to the slightly higher C content. Nonetheless, since the climate effect on growth may first be seen after long-term exposure that will reveal the contributing role of the belowground biomass to the perennial growth of the species, CO_2_ and temperature interactions are very important aspects of future climate change for the two investigated *P. australis* clones. The general observation in our study was that the effects of temperature were diminished under elevated CO_2_. The quantum yield (*α*), PNUE and some photosynthetic pigments were low at elevated temperature but only at ambient CO_2_. Hence, elevated CO_2_ seemed to offset the negative effects of elevated temperature.

## Conclusions and forward look

In conclusion, although in the short term a future rise in atmospheric CO_2_ to 700 ppm was found to barely affect the aboveground growth of the two *P. australis* clones investigated, most of their photosynthetic parameters responded positively to elevated CO_2_, and their SLA decreased as a commonly observed response to elevated CO_2_. Elevated temperature produced an increase in aboveground growth, regardless of the atmospheric CO_2_ concentration, suggesting that the ubiquitous range of *P. australis* will persist in the foreseeable warmer world. Further studies of total biomass production as well as biomass allocation to specific plant parts are needed to elucidate the overall growth responses of *P. australis* to a changed climate. The responses to temperature and CO_2_ concentration investigated here depended on the genetic background of the plant. The DK clone showed the strongest responses and may, due to its higher plasticity, be the clone better adapted to climate change. As our study shows that distinct *P. australis* clones respond dissimilarly to climate factors, in the future it would be worth investigating the responses of other clones of the same phylogenetic background that have a significant impact on their ecosystems. This would, for instance, be appropriate for native and invasive clones of the species, especially in North America, where co-occurring clones are competing and the invasive *Phragmites* threaten the ecosystem diversity.

## Sources of funding

This research was funded by the Danish Council for Independent Research—Natural Sciences, via a grant to H.B., and Aarhus Graduate School of Science.

## Contributions by the authors

F.E. carried out the experiment and drafted the manuscript. H.B., C.L., L.X.N. and L.A. participated in the design of the study. All authors helped to draft the manuscript and read and approved the final manuscript.

## Conflict of interest statement

None declared.
